# Inferring Retinal Degeneration-Related Genes Based on Xgboost

**DOI:** 10.3389/fmolb.2022.843150

**Published:** 2022-02-11

**Authors:** Yujie Xia, Xiaojie Li, Xinlin Chen, Changjin Lu, Xiaoyi Yu

**Affiliations:** ^1^ Department of Ophthalmology, The First Affiliated Hospital of Guangzhou University of Chinese Medicine, Guangzhou, China; ^2^ Guangzhou University of Chinese Medicine, Guangzhou, China

**Keywords:** retinitis degeneration, Xgboost, amino acids, pathogenic gene, machine learning

## Abstract

Retinal Degeneration (RD) is an inherited retinal disease characterized by degeneration of rods and cones photoreceptor cells and degeneration of retinal pigment epithelial cells. The age of onset and disease progression of RD are related to genes and environment. At present, research has discovered five genes closely related to RD. They are RHO, PDE6B, MERTK, RLBP1, RPGR, and researchers have developed corresponding gene therapy methods. Gene therapy uses vectors to transfer therapeutic genes, genetically modify target cells, and correct or replace disease-causing RD genes. Therefore, identifying the pathogenic genes of RD will play an important role in the development of treatment methods for the disease. However, the traditional methods of identifying RD-related genes are mostly based on animal experiments, and currently only a small number of RD-related genes have been identified. With the increase of biological data, Xgboost is purposed in this article to identify RP-related genes. Xgboost adds a regular term to control the complexity of the model, hence using Xgboost to find out true RD-related genes from complex and massive genes is suitable. The problem of overfitting can be avoided to some extent. To verify the power of Xgboost to identify RD-related genes, we did 10-cross validation and compared with three traditional methods: Random Forest, Back Propagation network, Support Vector Machine. The accuracy of Xgboost is 99.13% and AUC is much higher than other three methods. Therefore, this article can provide technical support for efficient identification of RD-related genes and help researchers have a deeper the understanding of the genetic characteristics of RD.

## Introduction

Hereditary eye diseases include syndromes and non-syndromic forms of retinal degeneration, hereditary glaucoma, corneal dystrophy and eye movement disorders. Retinal degeneration (RD) is a group of single-gene hereditary blindness caused by loss of function of photoreceptor cells or retinal pigment epithelium (RPE). The incidence of RDs worldwide is 1/3,000–1/2,000 (Berger et al., 2010). According to whether they are accompanied by systemic symptoms, they are divided into simple and systemic RDs (Wennström et al., 2003).The former mainly includes retinitis pigmentosa (RP), Rod cell dystrophy (cone-rod dystrophies, CORD), Leber congenital amaurosis (Leber congenital amaurosis, LCA), etc. The latter mainly includes Usher syndrome and Bardet-Biedl syndrome (Muller et al., 2010).Up to now, more than 300 pathogenic genes have been reported for RD, which suggests that RD has a high degree of clinical and genetic heterogeneity, the diagnosis of this type of disease is extremely difficult (Benayoun et al., 2009). Research on the pathogenic genes of RDs and the development and application of related molecular diagnostic techniques are the prerequisites for the diagnosis, prevention and treatment of RDs. Both single-gene Mendelian or complex hereditary eye diseases require genetic testing to determine the underlying cause. There are nearly 1,200 genes related to eye diseases in the human online Mendelian genetic database (on-line Mendelian inheritancein man, oMIM) (http://www.omim.org)(Amberger et al., 2015). RD is a type of disease with obvious clinical phenotypic heterogeneity and genetic heterogeneity, and it is also the main type of ophthalmic genetic diseases and rare and difficult ophthalmic diseases. At present, the vast majority of RD is still incurable in ophthalmology, and research on its diagnosis and treatment has always been a hot spot. Diagnosing RD at the genetic level is helpful for a deep understanding of the disease mechanism (Boycott et al., 2017). Distinguishing what kind of gene mutation causes the disease can more accurately understand the occurrence, development and outcome of the disease. This is especially important for RD with obvious heterogeneity. The genetic heterogeneity of RD requires a new disease naming and definition system. The system should include at least two main factors, namely the disease-causing gene and the name of the disease related to it. For example, EYS-related retinitis pigmentosa is more accurate than retinitis pigmentosa alone, and it is easier to explain the condition to the patient.

Because of the large number of pathogenic genes of retinal degeneration and the different mutation genes and loci in different families, it is very difficult to selectively screen candidate pathogenic genes. At present, the research on molecular genetics of hereditary eye disease is mainly family single gene research, which leads to controversy and deficiency in the genetic research of RD gene (Fan et al., 2006). A comprehensive and systematic analysis of known gene variation data may be helpful for the further study of such problems. Genes and mutations associated with retinal degeneration are controversial. Some genes were first reported to be disease-related, and then no mutations were reported. Although a large number of mutations in retinal degeneration are concentrated in a few genes, and the mutations of many genes only explain the causes of a very small number of patients, it is possible that only a very small number of patients with this gene carry mutations, but it cannot be ruled out that the previous research only found changes in a single gene and mistakenly believed that it was the cause of the disease. The controversial and questionable problems such as mutation penetrance and related risk factors reported in single gene research also bring confusion to researchers. In addition, because there was no public database containing a large number of variation data and a large number of control validation, some high-frequency SNPs were found in patients and were regarded as pathogenic mutations. These mutations are listed in the human gene mutation database (HGMD) as pathogenic mutations (Stenson et al., 2020), which mislead the follow-up molecular genetics research. At present, the reported variation analysis doubts and corrects the pathogenicity of individual Retnet genes and mutations (Pozo et al., 2015), such as the previously reported pathogenic genes fscn2 (MIM: 607643) and or2w3 of retinitis pigmentosa and hmcn1 (MIM: 608548) of macular degeneration (Fisher et al., 2007; Zhang et al., 2007; Sharon et al., 2016), and the subsequent research reports are questionable, but due to the lack of clinical phenotype analysis of patients with the same mutation, It is still impossible to completely deny its possibility as a pathogenic gene. In addition, single-gene research cannot comprehensively and systematically understand the genetic mutation spectrum of the people with hereditary retinal degeneration of this ethnic group. Different races have different gene mutation spectrums. Common disease-causing gene mutations in European and American populations are not common in Asian populations; based on common gene mutations in Asian populations, they may be very rare in European and American populations. For example, the pathogenic gene CNGA3 (MIM: 600053) of pyramidal cell dystrophy is the most frequently mutated gene in Chinese patients (Huang et al., 2016), and the most common recessive genetic mutation in foreign reports is ABCA4 (MIM: 601691) (Maugeri et al., 2000), CNGA3 only explains a small part of the cause of the disease (Wissinger et al., 2001). Even the Asian population has a different mutation spectrum. The highest mutation frequency in the Japanese retinitis pigmentosa population is EYS (MIM: 612424)(Oishi et al., 2014; Arai et al., 2015), and this gene mutation is very rare in Chinese patients (Xu et al., 2014; Chen et al., 2015). It is very important and necessary to conduct a comprehensive multi-gene systematic analysis of all retinal degeneration genes, and to understand the clinical characteristics, gene mutation frequency spectrum and discover the main pathogenic genes of the people with retinal degeneration of this nation. At the same time, it also provides important clinical evidence for the clinical diagnosis, genetic counseling, and prevention of hereditary eye diseases.

Although researchers have made great achievement in identifying RD-related genes, identifying the huge and complex acid sequences needs an algorithm which has high computational efficiency and high recognition accuracy. The generation of multi-omics data allows us to combine different data from a large number of samples to explore RD-related genes at a comprehensive level (Zhao et al., 2021a). Integrating multiple omics data to discover biological knowledge on a large scale has become a universal method. An endless stream of methods have been developed to apply to different research problems, such as identification of disease-related gene (Zhao et al., 2020; Antonarakis, 2021), identification of disease-related protein (Katako et al., 2018; Zhao et al., 2021b), identification of disease-related metabolite (Lei and Tie, 2019; Zhao et al., 2021c), disease-related drug target identification (Agamah et al., 2020; Zhao et al., 2021d), etc. Chen (Chen and Guestrin, 2016) purposed a novel method named Extreme Gradient Boosting (Xgboost) in 2004. He improved the boosting algorithm. Its multi-threaded parallel and regularization term not only improve the accuracy of the algorithm but also reduce the running time. Therefore, Xgboost is a suitable algorithm to solve the problem of identifying RD-related genes.

## Methods and Materials

### Data Description

We searched RD-related genes from DisGeNET (Piñero et al., 2020) by the key word “Retinal Degeneration.” There are 207 genes which are known to be related to RD in this database. We downloaded the sequences of these genes corresponding proteins from Uniprot (Consortium, 2019).

We also obtained 5,000 genes as genes potentially associated with RD from Genecard (Safran et al., 2010). Our aim is to identify RD-related genes from these 5,000 genes.

### Feature Extraction

#### Compositional Analysis

Since the real constitution of RD-related genes encoded proteins is quite different from the non-related genes’, the frequency of the occurrence of the all 20 amino acids in these proteins could be quite different.

We totally calculated the average amino acid composition of 207 RD-related genes encoded proteins. These proteins are richest in “L,” and the composition of “G,” “A,” “V,” “E,” “S” is very high.

#### Dissociation Constant

The protein structure is significantly related to the chemical characteristic of amino acid, especially hydrophobic and hydrophilic (Aftabuddin and Kundu, 2007). Aftabuddin et al. divided 20 amino acids into six groups based on the ranges of the hydropathy. The reason why the gene is related to RD is significantly related to the function of the protein it encodes. Therefore, the hydrophilicity and hydrophobicity of amino acids in protein are the key to judging whether the gene is related to RD. Table 1 shows the six groups of the 20 amino acids.

**TABLE 1:** The six groups of the 20 amino acids.

Groups	Amino acids
Strongly hydrophilic	R,D,E,N,Q,K,H
Strongly hydrophobic	L,I,V,A,M,F
Weakly hydrophilic or Weakly hydrophobic	S,T,Y,W
Proline	P
Glycine	G
Cysteine	C

So, the sequence of every protein could be diverted to a 6-dimension sequence. Each dimension is the average composition of one of these six groups.

#### PEST Regions

In 1986, Rechsteiner M and Rogers SW (Rechsteiner et al., 1996) made the assumption that the amino acids of “P,” “E,” “S” and “T” can serve as proteolytic signals. Now more and more reports have verified that the sequence which contains PEST regions can cause the rapid degradation of proteins.

The Epestfind program can be used to identify all poor and potential PEST protein sequences. (Espreafico et al., 1992) http://emboss.bioinformatics.nl/cgi-bin/emboss/epestfind.

We only included potential PEST protein region as a feature to identify the RD-related genes. We counted the number of potential pest regions in each sequence.

**FIGURE 1:**
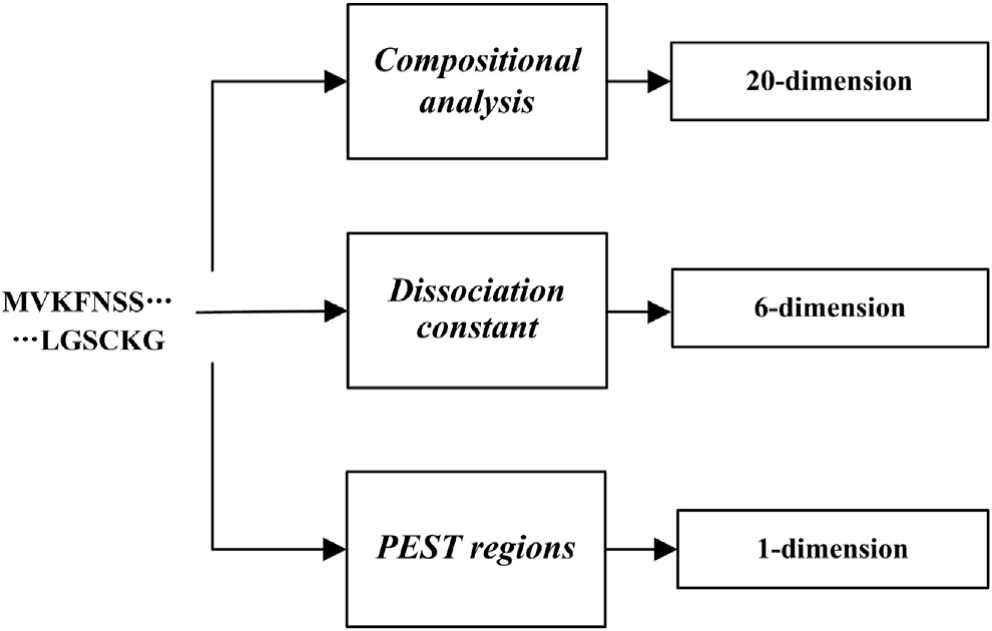
Flow chart of Feature extraction.

In conclusion, we totally extracted three kinds of features (Figure 1).

So, we used these 27-dimensions to identify the RD-related.

### Methods and Framework

#### Extreme Gradient Boosting

The Extreme Gradient Boosting (Xgboost) is the improvement of traditional Gradient Boosting Decision Tree (GBDT). Xgboost implements the first and the two order derivatives from the loss function by applying two order Taylor expansion. However, the traditional GBDT algorithm only implements first derivative information during optimizing. Xgboost runs significantly faster than GBDT. Because it has two advantages. On the one hand, Xgboost supports automatic multi-core parallel computing through open MP. On the other hand, Xgboost proposes a new data format Dmatrix, which can be preprocessed first and then trained. This improves the efficiency of each iteration of the training process and reduces the model training time. In addition, we can input the sparse matrix into xgboost.

First, we need to obtain our train set ${\left\{x_{i},y_{i}\right\}}^{N}$ , $y_{i}\in \left\{-1,1\right\}$ and set the number of leaf nodes as J. Then, we need to initialize the final function.

$$F_{0}\left(x\right)=\frac{1}{2}\log\frac{1+\bar{y}}{1-\bar{y}}$$ (1)

Then, the gradient of training samples can be obtained by:

$${\overset{⌢}{y}}_{i}=-\frac{\partial L\left(y_{i},F\left(x_{i}\right)\right)}{\partial F\left(x_{i}\right)}$$ (2)

Then, the CART regression tree ${\left\{R_{jm}\right\}}^{J}$ can be constructed. $R_{jm}$ is the j_th_ feature space.

Then, each leaf node’s regression value can be obtained by:

$$r_{jm}=\frac{\sum_{x_{i}\in R_{jm}}{\overset{⌢}{y}}_{i}}{\sum_{x_{i}\in R_{jm}}\left|{\overset{⌢}{y}}_{i}\right|\left(2-\left|{\overset{⌢}{y}}_{i}\right|\right)}$$ (3)

Finally, the final model is as following:

$$F_{m}\left(x\right)=F_{m-1}\left(x\right)+\sum_{j=1}^{J}r_{jm}I\left(x\in R_{jm}\right)$$ (4)

The objective function is consisted by loss function and regularization term, which can be used to show the quality of our method.

Obj(Θ)=L(θ)+Ω(Θ) (5)

L(θ) represents loss function. Algorithms such as artificial neural networks only use loss function to evaluate the quality of training, which is easy to cause over fitting. The regularization parameters Ω(Θ) are introduced into methods such as support vector machine, which can effectively reduce over fitting. However, the introduction of regularization parameters will increase the complexity of the model.

CART is the basic unit of Xgboost. Therefore, the objective function in formula (5) can also be represented as following:

$$Obj\left(\Theta \right)=\sum_{i}^{n}l\left(y_{i},{\overset{⌢}{y}}_{i}\right)+\sum_{t=1}^{T}\Omega \left(f_{t}\right)$$ (6)

Each tree is obtained based on the last tree we constructed.

$$\begin{array}{l} {\overset{⌢}{y}}_{i}^{0}=0,\\ {\overset{⌢}{y}}_{i}^{1}=f_{1}\left(x_{i}\right)={\overset{⌢}{y}}_{i}^{0}+f_{1}\left(x_{i}\right),\\ {\overset{⌢}{y}}_{i}^{2}=f_{1}\left(x_{i}\right)+f_{2}\left(x_{i}\right)={\overset{⌢}{y}}_{i}^{1}+f_{2}\left(x_{i}\right),\\ \vdots \\ {\overset{⌢}{y}}_{i}^{2}=\sum_{k=1}^{t}f_{k}\left(x_{i}\right)={\overset{⌢}{y}}_{i}^{t-1}+f_{t}\left(x_{i}\right), \end{array}$$ (7)

Finally, we can obtained the first and the two order derivatives from the loss function.

$$Obj^{\left(t\right)}=\sum_{i}^{n}\left(l\left(y_{i},{\overset{⌢}{y}}_{i}^{t-1}\right)+g_{i}f_{t}\left(x_{i}\right)+\frac{1}{2}h_{i}f_{t}^{2}\left(x_{i}\right)\right)+\Omega \left(f_{t}\right)+\mathrm{constant}$$ (8)

The next part is to obtain regularization term. Firstly, we define the decision tree as:

$$f_{t}\left(x\right)=w_{q\left(x\right)},w\in R^{M},q:R^{d}\to \left\{1,2,\cdots ,M\right\}$$ (9)

w represents leaf node’s score. q(x) is used to determine the position of the input sample in the tree. The regularization term can be represented as following:

$$\Omega \left(f\right)=\gamma M+\frac{1}{2}\lambda \sum_{j=1}^{M}w_{j}^{2}$$ (10)

We need to set γ and λ to balance the complexity of the model.

So t_th_ tree’s objective function is as following:

$$\begin{array}{c} Obj^{\left(t\right)}\approx \sum_{i=1}^{n}\left(g_{i}w_{q}\left(x_{i}\right)+\frac{1}{2}h_{i}w_{q}^{2}\left(x_{i}\right)\right)+\gamma M+\frac{1}{2}\lambda \sum_{j=1}^{M}w_{j}^{2}\\ =\sum_{j=1}^{M}\left((\sum g_{i}w_{j}+\frac{1}{2}\left(\sum h_{i}+\lambda \right)w_{j}^{2}\right)+\gamma M \end{array}$$ (11)

We could define $G_{j}=\sum g_{i}$ and $H_{j}=\sum h_{i}$ , then we get:

$$Obj^{\left(t\right)}=\sum_{j=1}^{M}\left(G_{j}w_{j}+\frac{1}{2}\left(H_{j}+\lambda \right)w_{j}^{2}\right)+\gamma M$$ (12)

## Results

### Experiment Description

We totally got 207 true RD-related genes and we randomly selected 5,000 genes as the negative samples. To verify the effectiveness of Xgboost on identifying RD-related genes, we did ten-cross validation.

We randomly divided these 5,207 sequences into ten groups. For every group, we choose 520 sequences as the test set and the rest 4,687 sequences as the train set. So, we did ten experiments in total. Besides, every sequence has become a training set and a test set. We set the parameters of Xgboost as the Table 2.

**TABLE 2:** The parameters of the Xgboost.

Setting items	The value set
Booster	gbtree
Silent	0
Learning rate	0.3
Maximum depth of a tree	6
Minimum sum of instance weight	1
Subsample ratio	1
Experimental parameter	1

### Evaluation Criteria

We use four evaluation ways to evaluate the performance of Xgboost on identifying RD-related genes.

We put the results of the ten experiments in the Table 2. A total of 5,207 sequences were tested. As showed in Table 3, we could calculate the Accuracy = 99.13%, Precision = 99.04%, Recall = 99.23%, Specificity = 99.04%.

**TABLE 3:** The results of the ten experiments.

		Prediction		
		1	0	Total
True Label	1	205 (TP)	2(FN)	207
	0	20(FP)	4,980 (TN)	5,000
Total		225	4,982	5,207

### Experiments Result

In this study, the label of randomly selected genes is 0, and the label of RD-related genes are 1.

**FIGURE 2:**
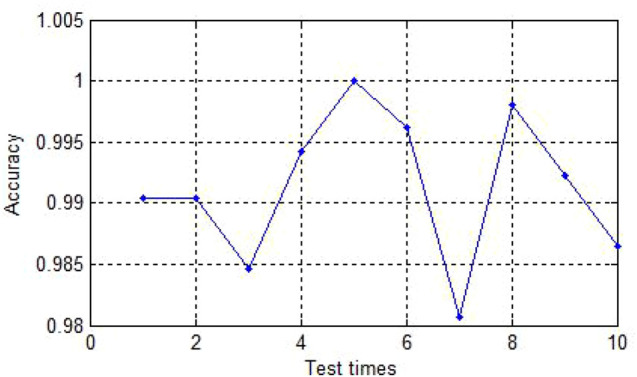
The accuracy of ten experiments.

The Figure 2 shows the curves of the ten times experiments’ accuracy. As we can see, the experiment with the lowest accuracy is also more than 98%.

To verify the superiority of the Xgboost, we also use the same data to do the ten-cross validation by other methods. We use Back Propagation network (BP), Random Forest (RF), Support Vector Machine (SVM) respectively. The error statistics of the average results of 10 experiments are shown in the following table.

As we can see in the Table 4, we could see the performance of Xgboost is the best, and the performance of BP is the worst. Although RF is better than the Xgboost in the evaluations of ‘Precision’ and “Specificity,” the accuracy of the Xgboost is the best. Besides, Xgboost uses the least time to build up the model.

**TABLE 4:** Comparison of the Xgboost with alternative models.

Algorithm	ACC (%)	Precision (%)	Recall (%)	Specificity (%)
Xgboost	99.13	99.04	99.23	99.04
BP	82.50	78.13	90.25	74.76
Random Forest	97.99	99.64	96.34	99.65
SVM	94.16	94.62	93.64	94.68

**FIGURE 3:**
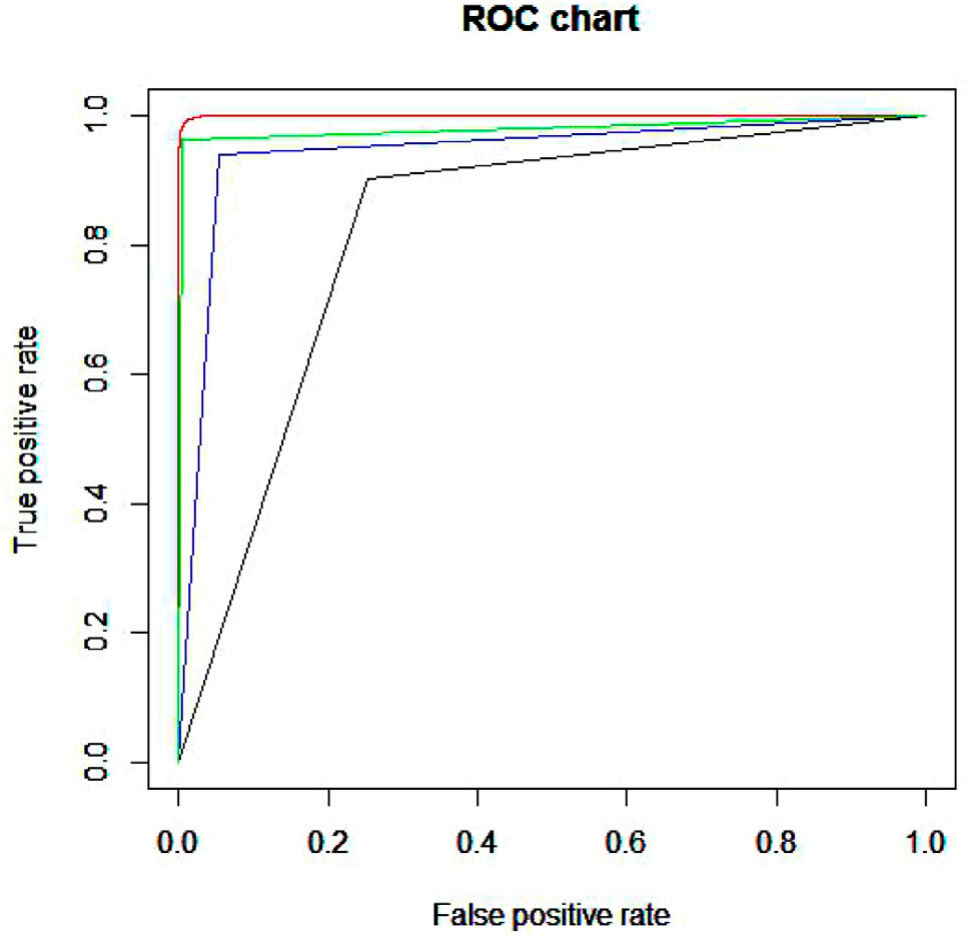
ROC curve of four methods.

**FIGURE 4:**
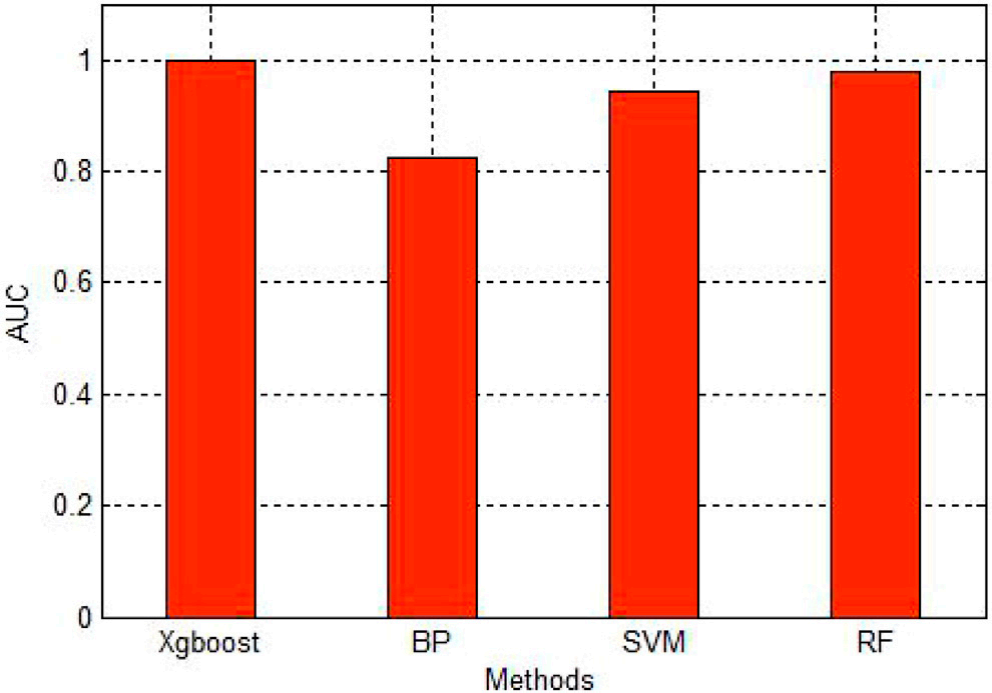
AUC of four methods.

Figure 3 is the ROC curve of four methods. The red line is the curve of Xgboost. The green line is the curve of RF. The blue and black one is the SVM and BP respectively. As we can see in the figure, Xgboost is the best among these four methods. Then we draw a figure of AUC in the Figure 4.

As we can see in the Figure 4, the AUC of Xgboost is very close to 1. It shows the high accuracy of the Xgboost.

## Conclusion

Typical clinical features of RD include early night blindness, subsequent progressive vision loss and narrowing of the visual field, fundus showing osteocytic pigmentation, waxy pale atrophy of the optic disc, and electroretinogram (ERG) cone and rod Cell function decline, etc., the early rod cell response amplitude decline is more serious than the cone cell response amplitude. Due to the high degree of heterogeneity of the RP phenotype, many retinopathy have similar symptoms with RP, which is very easy to confuse.

Therefore, exploring RD from a genetic perspective is very helpful for clinical diagnosis, treatment and research on the pathogenic mechanism of diseases. With the popularization of high-throughput sequencing technology, a large amount of genome and proteomic data has been released. However, no method has been proposed to specifically identify RD-related genes. In this article, we propose a method based on XGboost to identify RD-related genes. We extracted three features of the corresponding proteins of 207 genes known to be related to RD. Each gene has 27-dimensional features, and we input these features into Xgboost for training. Through 10-fold cross-validation, we confirmed the accuracy of our method to identify RD-related genes with AUC as 0.99.

In summary, we propose a method for large-scale identification of RD-related genes. This type of machine learning method can prioritize genes that are potentially related to RD to save researchers the cost of conducting biological experiments.

## Data Availability

The datasets presented in this study can be found in online repositories. The names of the repository/repositories and accession number(s) can be found in the article/Supplementary Material.

## References

[B1] Aftabuddin M. Kundu S. (2007). Hydrophobic, Hydrophilic, and Charged Amino Acid Networks within Protein. Biophysical J. 93 (1), 225–231. 10.1529/biophysj.106.098004 PMC1914426 17172302

[B2] Agamah F. E. Mazandu G. K. Hassan R. Bope C. D. Thomford N. E. Ghansah A. (2020). Computational/In Silico Methods in Drug Target and lead Prediction. Brief. Bioinformatics 21 (5), 1663–1675. 10.1093/bib/bbz103 31711157 PMC7673338

[B3] Amberger J. S. Bocchini C. A. Schiettecatte F. Scott A. F. Hamosh A. (2015). OMIM.org: Online Mendelian Inheritance in Man (OMIM), an Online Catalog of Human Genes and Genetic Disorders. Nucleic Acids Res. 43 (D1), D789–D798. 10.1093/nar/gku1205 25428349 PMC4383985

[B4] Antonarakis S. E. (2021). History of the Methodology of Disease Gene Identification. Hoboken, New Jersey, United States: Wiley Online Library. 10.1002/ajmg.a.62400 PMC8596769 34159713

[B5] Arai Y. Maeda A. Hirami Y. Ishigami C. Kosugi S. Mandai M. (2015). Retinitis Pigmentosa with EYS Mutations Is the Most Prevalent Inherited Retinal Dystrophy in Japanese Populations. J. Ophthalmol. 2015, 819760. 10.1155/2015/819760 26161267 PMC4487330

[B6] Benayoun L. Spiegel R. Auslender N. Abbasi A. H. Rizel L. Hujeirat Y. (2009). Genetic Heterogeneity in Two Consanguineous Families Segregating Early Onset Retinal Degeneration: the Pitfalls of Homozygosity Mapping. Am. J. Med. Genet. 149A (4), 650–656. 10.1002/ajmg.a.32634 19140180

[B7] Berger W. Kloeckener-Gruissem B. Neidhardt J. (2010). The Molecular Basis of Human Retinal and Vitreoretinal Diseases. Prog. Retin. Eye Res. 29 (5), 335–375. 10.1016/j.preteyeres.2010.03.004 20362068

[B8] Boycott K. M. Rath A. Chong J. X. Hartley T. Alkuraya F. S. Baynam G. (2017). International Cooperation to Enable the Diagnosis of All Rare Genetic Diseases. Am. J. Hum. Genet. 100 (5), 695–705. 10.1016/j.ajhg.2017.04.003 28475856 PMC5420351

[B9] Chen T. Guestrin C. “XGBoost: A Scalable Tree Boosting System,” in Proceedings of the 22nd ACM SIGKDD International Conference on Knowledge Discovery and Data Mining, San Francisco California USA, August 2016, 785–794.

[B10] Chen X. Liu X. Sheng X. Gao X. Zhang X. Li Z. (2015). Targeted Next-Generation Sequencing Reveals Novel EYS Mutations in Chinese Families with Autosomal Recessive Retinitis Pigmentosa. Sci. Rep. 5 (1), 8927. 10.1038/srep08927 25753737 PMC4354143

[B11] Consortium U. (2019). UniProt: a Worldwide Hub of Protein Knowledge. Nucleic Acids Res. 47 (D1), D506–D515. 10.1093/nar/gky1049 30395287 PMC6323992

[B12] Espreafico E. M. Cheney R. E. Matteoli M. Nascimento A. A. De Camilli P. V. Larson R. E. (1992). Primary Structure and Cellular Localization of Chicken Brain Myosin-V (P190), an Unconventional Myosin with Calmodulin Light Chains. J. Cel Biol. 119 (6), 1541–1557. 10.1083/jcb.119.6.1541 PMC2289763 1469047

[B13] Fan B. J. Tam P. O. S. Choy K. W. Wang D. Y. Lam D. S. C. Pang C. P. (2006). Molecular Diagnostics of Genetic Eye Diseases. Clin. Biochem. 39 (3), 231–239. 10.1016/j.clinbiochem.2005.11.010 16412407

[B14] Fisher S. A. Rivera A. Fritsche L. G. Keilhauer C. N. Lichtner P. Meitinger T. (2007). Case-control Genetic Association Study of Fibulin-6 (FBLN6orHMCN1) Variants in Age-Related Macular Degeneration (AMD). Hum. Mutat. 28 (4), 406–413. 10.1002/humu.20464 17216616

[B15] Huang L. Xiao X. Li S. Jia X. Wang P. Sun W. (2016). Molecular Genetics of Cone-Rod Dystrophy in Chinese Patients: New Data from 61 Probands and Mutation Overview of 163 Probands. Exp. Eye Res. 146, 252–258. 10.1016/j.exer.2016.03.015 26992781

[B16] Katako A. Shelton P. Goertzen A. L. Levin D. Bybel B. Aljuaid M. (2018). Machine Learning Identified an Alzheimer's Disease-Related FDG-PET Pattern Which Is Also Expressed in Lewy Body Dementia and Parkinson's Disease Dementia. Sci. Rep. 8 (1), 13236. 10.1038/s41598-018-31653-6 30185806 PMC6125295

[B17] Lei X. Tie J. (2019). Prediction of Disease-Related Metabolites Using Bi-random Walks. PloS one 14 (11), e0225380. 10.1371/journal.pone.0225380 31730648 PMC6857945

[B18] Maugeri A. Klevering B. J. Rohrschneider K. Blankenagel A. Brunner H. G. Deutman A. F. (2000). Mutations in the ABCA4 (ABCR) Gene Are the Major Cause of Autosomal Recessive Cone-Rod Dystrophy. Am. J. Hum. Genet. 67 (4), 960–966. 10.1086/303079 10958761 PMC1287897

[B19] Muller J. Stoetzel C. Vincent M. C. Leitch C. C. Laurier V. Danse J. M. (2010). Identification of 28 Novel Mutations in the Bardet-Biedl Syndrome Genes: the burden of Private Mutations in an Extensively Heterogeneous Disease. Hum. Genet. 127 (5), 583–593. 10.1007/s00439-010-0804-9 20177705 PMC3638942

[B20] Oishi M. Oishi A. Gotoh N. Ogino K. Higasa K. Iida K. (2014). Comprehensive Molecular Diagnosis of a Large Cohort of Japanese Retinitis Pigmentosa and Usher Syndrome Patients by Next-Generation Sequencing. Invest. Ophthalmol. Vis. Sci. 55 (11), 7369–7375. 10.1167/iovs.14-15458 25324289

[B21] Piñero J. Ramírez-Anguita J. M. Saüch-Pitarch J. Ronzano F. Centeno E. Sanz F. (2020). The DisGeNET Knowledge Platform for Disease Genomics: 2019 Update. Nucleic Acids Res. 48 (D1), D845–D855. 10.1093/nar/gkz1021 31680165 PMC7145631

[B22] Pozo M. G. Bravo-Gil N. Méndez-Vidal C. Montero-de-Espinosa I. Millán J. M. Dopazo J. (2015). Re-evaluation Casts Doubt on the Pathogenicity of Homozygous USH2A p.C759F. Am. J. Med. Genet. A. 167 (7), 1597–1600. 10.1002/ajmg.a.37003 25823529

[B23] Rechsteiner M. Rogers S. W. “Rechsteiner M. Rogers S. W. (1996). PEST Sequences and Regulation by Proteolysis. Trends Biochem. Sci. 2121 (7), 267267–271271. 10.1016/s0968-0004(96)10031-1 8755249

[B24] Safran M. Dalah I. Alexander J. Rosen N. Iny Stein T. Shmoish M. (2010). GeneCards Version 3: the Human Gene Integrator. Database (Oxford) 20102010, baq020. 10.1093/database/baq020 PMC2938269 20689021

[B25] Sharon D. Kimchi A. Rivolta C. (2016). OR2W3 Sequence Variants Are Unlikely to Cause Inherited Retinal Diseases. Ophthalmic Genet. 37 (4), 366–368. 10.3109/13816810.2015.1081252 26891008

[B26] Stenson P. D. Mort M. Ball E. V. Chapman M. Evans K. Azevedo L. (2020). The Human Gene Mutation Database (HGMD®): Optimizing its Use in a Clinical Diagnostic or Research Setting. Hum. Genet. 139 (10), 1197–1207. 10.1007/s00439-020-02199-3 32596782 PMC7497289

[B27] Wennström A. Ericson L. García-Guzmán G. (2003). The Concept of Sexually Transmitted Diseases in Plants: Definition and Applicability. Oikos 100 (2), 397–402. 10.1034/j.1600-0706.2003.12004.x

[B28] Wissinger B. Gamer D. Jägle H. Giorda R. Marx T. Mayer S. (2001). CNGA3 Mutations in Hereditary Cone Photoreceptor Disorders. Am. J. Hum. Genet. 69 (4), 722–737. 10.1086/323613 11536077 PMC1226059

[B29] Xu Y. Guan L. Shen T. Zhang J. Xiao X. Jiang H. (2014). Mutations of 60 Known Causative Genes in 157 Families with Retinitis Pigmentosa Based on Exome Sequencing. Hum. Genet. 133 (10), 1255–1271. 10.1007/s00439-014-1460-2 24938718

[B30] Zhang Q. Li S. Xiao X. Jia X. Guo X. (2007). The 208delG Mutation inFSCN2Does Not Associate with Retinal Degeneration in Chinese Individuals. Invest. Ophthalmol. Vis. Sci. 48 (2), 530–533. 10.1167/iovs.06-0669 17251446

[B31] Zhao T. Hu Y. Cheng L. (2021). Deep-DRM: a Computational Method for Identifying Disease-Related Metabolites Based on Graph Deep Learning Approaches. Brief Bioinform 22 (4), bbaa212. 10.1093/bib/bbaa212 33048110

[B32] Zhao T. Hu Y. Peng J. Cheng L. (2020). DeepLGP: a Novel Deep Learning Method for Prioritizing lncRNA Target Genes. Bioinformatics 36 (16), 4466–4472. 10.1093/bioinformatics/btaa428 32467970

[B33] Zhao T. Hu Y. Valsdottir L. R. Zang T. Peng J. (2021). Identifying Drug-Target Interactions Based on Graph Convolutional Network and Deep Neural Network. Brief. Bioinformatics 22 (2), 2141–2150. 10.1093/bib/bbaa044 32367110

[B34] Zhao T. Liu J. Zeng X. Wang W. Li S. Zang T. (2021). Prediction and Collection of Protein–Metabolite Interactions. Brief. Bioinform. 22, bbab014. 10.1093/bib/bbab014 33554247

[B35] Zhao T. Lyu S. Lu G. Juan L. Zeng X. Wei Z. (2021). SC2disease: a Manually Curated Database of Single-Cell Transcriptome for Human Diseases. Nucleic Acids Res. 49 (D1), D1413–D1419. 10.1093/nar/gkaa838 33010177 PMC7778914

